# Supplementary material for: Maternally transmitted anti-measles antibodies, and susceptibility to disease among infants in Chandigarh, India: A prospective birth cohort study
Source: PLoS One. 2023 Oct 3;18(10):e0287110. doi: 10.1371/journal.pone.0287110 (PMC10547151; doi:10.1371/journal.pone.0287110)
Supplement: Attachment — Submitted filename: Response to Reviewers.docx [file pone.0287110.s004.docx]

PONE-D-22-30962R2

Maternally transmitted anti-measles antibodies, and susceptibility to disease among infants in Chandigarh, India:  A prospective birth cohort study.

Response to Editorial and Reviewers’ comments

We are grateful to the Editor and the Reviewers for their careful re-appraisal of our manuscript. We have glad that the reviewers have accepted the revised manuscript, and that two minor revisions have been suggested. We have considered both comments carefully and addressed them.

Please find enclosed herewith:

1) A detailed point by point response to the comments and suggestions offered.
2) Revised manuscript with Track changes. All newly added text is also highlighted in blue color for easy identification.
3) Manuscript (revised manuscript clean copy).

We hope that the revised manuscript is acceptable to be considered for publication.

Thanking you,

Yours sincerely,

Joseph L. Mathew

29 May 2023

PLOS ONE

Dear Dr. Mathew,

Thank you for submitting your manuscript to PLOS ONE. After careful consideration, we feel that it has merit but does not fully meet PLOS ONE’s publication criteria as it currently stands. Therefore, we invite you to submit a revised version of the manuscript that addresses the points raised during the review process.

Please submit your revised manuscript by Jun 29 2023 11:59PM. If you will need more time than this to complete your revisions, please reply to this message or contact the journal office at [plosone@plos.org](mailto:plosone@plos.org). When you're ready to submit your revision, log on to <https://www.editorialmanager.com/pone/> and select the 'Submissions Needing Revision' folder to locate your manuscript file.

Please include the following items when submitting your revised manuscript:

- A rebuttal letter that responds to each point raised by the academic editor and reviewer(s). You should upload this letter as a separate file labeled 'Response to Reviewers'.
- A marked-up copy of your manuscript that highlights changes made to the original version. You should upload this as a separate file labeled 'Revised Manuscript with Track Changes'.
- An unmarked version of your revised paper without tracked changes. You should upload this as a separate file labeled 'Manuscript'.

**Authors’ response**: We have followed the instructions given above, and enclosed the three files as specified.

If you would like to make changes to your financial disclosure, please include your updated statement in your cover letter. Guidelines for resubmitting your figure files are available below the reviewer comments at the end of this letter.

**Authors’ response**: There is no change to the financial disclosure statement.

If applicable, we recommend that you deposit your laboratory protocols in [protocols.io](http://protocols.io) to enhance the reproducibility of your results. Protocols.io assigns your protocol its own identifier (DOI) so that it can be cited independently in the future. For instructions see: <https://journals.plos.org/plosone/s/submission-guidelines#loc-laboratory-protocols>. Additionally, PLOS ONE offers an option for publishing peer-reviewed Lab Protocol articles, which describe protocols hosted on [protocols.io](http://protocols.io). Read more information on sharing protocols at <https://plos.org/protocols?utm_medium=editorial-email&utm_source=authorletters&utm_campaign=protocols>.

**Authors’ response**: Thank you for the suggestion. Standard laboratory methods and protocols were used. As there is no novel method used, we have cited the relevant references in the manuscript.

We look forward to receiving your revised manuscript.

Kind regards,

Ghada Abdrabo Abdellatif Elshaarawy, M.D
Academic Editor
PLOS ONE

Journal Requirements:

Please review your reference list to ensure that it is complete and correct. If you have cited papers that have been retracted, please include the rationale for doing so in the manuscript text, or remove these references and replace them with relevant current references. Any changes to the reference list should be mentioned in the rebuttal letter that accompanies your revised manuscript. If you need to cite a retracted article, indicate the article’s retracted status in the References list and also include a citation and full reference for the retraction notice.

**Authors’ response**: We have followed the instructions given above.

Additional Editor Comments:

Make sure you thoroughly address the reviewers’ comments and concerns and ensure the manuscript is free of any editorial or grammatical errors.

**Authors’ response**: We have followed the instructions given above, and done our best to ensure that the revised manuscript is free from errors.

[Note: HTML markup is below. Please do not edit.]

Reviewers' comments:

Reviewer's Responses to Questions

**Comments to the Author**

1. If the authors have adequately addressed your comments raised in a previous round of review and you feel that this manuscript is now acceptable for publication, you may indicate that here to bypass the “Comments to the Author” section, enter your conflict of interest statement in the “Confidential to Editor” section, and submit your "Accept" recommendation.

Reviewer #3: All comments have been addressed

Reviewer #4: All comments have been addressed

Reviewer #5: All comments have been addressed

**Authors’ response**: Thank you for confirming that all comments have been addressed.

2. Is the manuscript technically sound, and do the data support the conclusions?

The manuscript must describe a technically sound piece of scientific research with data that supports the conclusions. Experiments must have been conducted rigorously, with appropriate controls, replication, and sample sizes. The conclusions must be drawn appropriately based on the data presented.

Reviewer #3: Yes

Reviewer #4: Yes

Reviewer #5: Yes

**Authors’ response**: Thank you for the confirmation.

3. Has the statistical analysis been performed appropriately and rigorously?

Reviewer #3: Yes

Reviewer #4: Yes

Reviewer #5: Yes

**Authors’ response**: Thank you for the confirmation.

4. Have the authors made all data underlying the findings in their manuscript fully available?

The [PLOS Data policy](http://www.plosone.org/static/policies.action#sharing) requires authors to make all data underlying the findings described in their manuscript fully available without restriction, with rare exception (please refer to the Data Availability Statement in the manuscript PDF file). The data should be provided as part of the manuscript or its supporting information, or deposited to a public repository. For example, in addition to summary statistics, the data points behind means, medians and variance measures should be available. If there are restrictions on publicly sharing data—e.g. participant privacy or use of data from a third party—those must be specified.

Reviewer #3: Yes

Reviewer #4: Yes

Reviewer #5: Yes

**Authors’ response**: Thank you for the confirmation.

5. Is the manuscript presented in an intelligible fashion and written in standard English?

PLOS ONE does not copyedit accepted manuscripts, so the language in submitted articles must be clear, correct, and unambiguous. Any typographical or grammatical errors should be corrected at revision, so please note any specific errors here.

Reviewer #3: Yes

Reviewer #4: Yes

Reviewer #5: Yes

**Authors’ response**: Thank you for the confirmation.

6. Review Comments to the Author

Please use the space provided to explain your answers to the questions above. You may also include additional comments for the author, including concerns about dual publication, research ethics, or publication ethics. (Please upload your review as an attachment if it exceeds 20,000 characters)

Reviewer #3: The authors should do a few English corrections especially where words were omitted within a sentence eg line 135. The authors also need to adopt a uniform writing style either American or British eg the spelling of 'titer' or 'titre'.

**Authors’ response**: Thank you for pointing out the error. At all places, the word is spelt as “titer”.

**Revised text:**

In Line 135, the sentence has been changed as shown below:

Original sentence: Those were willing to participate…….

Revised sentence: Those who were willing to participate………

Reviewer #4: (No Response)

Reviewer #5: Substantial improvement of the manuscript, applying the appropriate data analysis and interpretation and solid conclusion of the research work.
Only the figure needs some enhancement (colors scheme) and layout. the time should be explicitly mentioned (0, 3. 6, 9 months)

**Authors’ response**:

Thank you for the positive comments.

As advised, the figure has been substantially modified with the following changes:

1. We did not fully understand the comment about the colors scheme. Therefore, we have uploaded the Figure in greyscale version as well as color version. The Editor in Chief is requested to choose whichever is better in appearance.
2. The time (0,3,6,9 months) has been explicitly mentioned.
3. The legend “Infant age in months (mo)” has been incorporated just beneath the ages.
4. The layout has been modified for greater clarity.
5. The Y axis on the right side has been re-formatted as per the scale of the Geometric Mean Titre (GMT).
6. The Y axis label “Geometric Mean Titre (GMT [IU/ml])” has been placed right next to the Y axis.

7. PLOS authors have the option to publish the peer review history of their article ([what does this mean?](https://journals.plos.org/plosone/s/editorial-and-peer-review-process#loc-peer-review-history)). If published, this will include your full peer review and any attached files.


If you choose “no”, your identity will remain anonymous but your review may still be made public.


**Do you want your identity to be public for this peer review?** For information about this choice, including consent withdrawal, please see our [Privacy Policy](https://www.plos.org/privacy-policy).

Reviewer #3: **Yes:** Dr Babatunde Adewale

Reviewer #4: **Yes:** Mohammad Ashraful Amin

Reviewer #5: **Yes:** Tarek Tawfik Amin

**Authors’ response**: Thank you for the confirmation.

The authors have no difficulty with the peer review history of the article being published.
