# Supplementary material for: Maternally transmitted anti-measles antibodies, and susceptibility to disease among infants in Chandigarh, India: A prospective birth cohort study
Source: PLoS One. 2023 Oct 3;18(10):e0287110. doi: 10.1371/journal.pone.0287110 (PMC10547151; doi:10.1371/journal.pone.0287110)
Supplement: Attachment — Submitted filename: RESPONSE TO REVIEWER COMMENTS.docx [file pone.0287110.s002.docx]

**Response to Editorial and Reviewers’ comments**

**We are grateful to the Editor and the Reviewers for their careful appraisal of our manuscript. We have considered each comment carefully and addressed each point. We believe that this has significantly enhanced the manuscript.**

**Please find enclosed herewith:**

1) **A detailed point by point response to the comments and suggestions offered.**
2) **Revised manuscript with Track changes. All newly added text is also highlighted in blue color for easy identification.**
3) **Revised manuscript (clean copy).**

**We hope that the revised manuscript is acceptable to be considered for publication.**

**Thanking you,**

**Yours sincerely,**

**Joseph L. Mathew**

**20 Feb 2023**

PlosOne REVIEWER COMMENTS

Dear Dr. Mathew,

Thank you for submitting your manuscript to PLOS ONE. After careful consideration, we feel that it has merit but does not fully meet PLOS ONE’s publication criteria as it currently stands. Therefore, we invite you to submit a revised version of the manuscript that addresses the points raised during the review process.

Please submit your revised manuscript by Feb 22, 2023, 11:59PM. If you will need more time than this to complete your revisions, please reply to this message or contact the journal office at [plosone@plos.org](mailto:plosone@plos.org). When you're ready to submit your revision, log on to <https://www.editorialmanager.com/pone/> and select the 'Submissions Needing Revision' folder to locate your manuscript file.

Please include the following items when submitting your revised manuscript:

- A rebuttal letter that responds to each point raised by the academic editor and reviewer(s). You should upload this letter as a separate file labeled 'Response to Reviewers'.
- A marked-up copy of your manuscript that highlights changes made to the original version. You should upload this as a separate file labeled 'Revised Manuscript with Track Changes'.
- An unmarked version of your revised paper without tracked changes. You should upload this as a separate file labeled 'Manuscript'.

If you would like to make changes to your financial disclosure, please include your updated statement in your cover letter. Guidelines for resubmitting your figure files are available below the reviewer comments at the end of this letter.

If applicable, we recommend that you deposit your laboratory protocols in [protocols.io](http://protocols.io) to enhance the reproducibility of your results. Protocols.io assigns your protocol its own identifier (DOI) so that it can be cited independently in the future. For instructions see: <https://journals.plos.org/plosone/s/submission-guidelines#loc-laboratory-protocols>. Additionally, PLOS ONE offers an option for publishing peer-reviewed Lab Protocol articles, which describe protocols hosted on [protocols.io](http://protocols.io). Read more information on sharing protocols at <https://plos.org/protocols?utm_medium=editorial-email&utm_source=authorletters&utm_campaign=protocols>.

We look forward to receiving your revised manuscript.

Kind regards,

Ghada Abdrabo Abdellatif Elshaarawy, M.D
Academic Editor
PLOS ONE

Journal Requirements:

When submitting your revision, we need you to address these additional requirements.

**Comment**

1. Please ensure that your manuscript meets PLOS ONE's style requirements, including those for file naming. The PLOS ONE style templates can be found at 
<https://journals.plos.org/plosone/s/file?id=wjVg/PLOSOne_formatting_sample_main_body.pdf> and <https://journals.plos.org/plosone/s/file?id=ba62/PLOSOne_formatting_sample_title_authors_affiliations.pdf>

**Response: Thank you for the advice. We have checked and confirm that the manuscript meets the requirements listed.**

**Comment**

2. Please ensure that you include a title page within your main document. We do appreciate that you have a title page document uploaded as a separate file, however, as per our author guidelines (<http://journals.plos.org/plosone/s/submission-guidelines#loc-title-page>) we do require this to be part of the manuscript file itself and not uploaded separately.
Could you therefore please include the title page into the beginning of your manuscript file itself, listing all authors and affiliations.

**Response: Thank you for the advice. The Title page has been included in the manuscript file.**

**Comment**

3. In your Data Availability statement, you have not specified where the minimal data set underlying the results described in your manuscript can be found. PLOS defines a study's minimal data set as the underlying data used to reach the conclusions drawn in the manuscript and any additional data required to replicate the reported study findings in their entirety. All PLOS journals require that the minimal data set be made fully available. For more information about our data policy, please see <http://journals.plos.org/plosone/s/data-availability>.

Upon re-submitting your revised manuscript, please upload your study’s minimal underlying data set as either Supporting Information files or to a stable, public repository and include the relevant URLs, DOIs, or accession numbers within your revised cover letter. For a list of acceptable repositories, please see <http://journals.plos.org/plosone/s/data-availability#loc-recommended-repositories>. Any potentially identifying patient information must be fully anonymized.

Important: If there are ethical or legal restrictions to sharing your data publicly, please explain these restrictions in detail. Please see our guidelines for more information on what we consider unacceptable restrictions to publicly sharing data: <http://journals.plos.org/plosone/s/data-availability#loc-unacceptable-data-access-restrictions>. Note that it is not acceptable for the authors to be the sole named individuals responsible for ensuring data access.

We will update your Data Availability statement to reflect the information you provide in your cover letter.

**Response: The datasets have been submitted along with the revised manuscript.**

**Comment**

4. We note that you have stated that you will provide repository information for your data at acceptance. Should your manuscript be accepted for publication, we will hold it until you provide the relevant accession numbers or DOIs necessary to access your data. If you wish to make changes to your Data Availability statement, please describe these changes in your cover letter and we will update your Data Availability statement to reflect the information you provide.

**Response: The datasets have been submitted along with the revised manuscript.**

**Additional Editor Comments:**

**Comment**
1) ABSTRACT:
¬ A brief background, plus the aim, should be added.

**Response: Thank you for the advice. This has been added.**

**Revised text:**

Prior to the age of measles vaccination, infants are believed to be protected against measles by passively transferred maternal antibodies. However, the quantity and quality of such protection have not been well established. We undertook this study to characterize the transfer and decline in maternal anti-measles antibodies among infants, and determine their susceptibility to measles. In this population-based, birth-cohort study, we enrolled pregnant women and their newborn infants, from a catchment area of 30 anganwadis in Chandigarh, India. We collected maternal blood at delivery, and infant samples at birth, and 3, 6, and 9 months of age. Anti-measles IgG antibodies were measured using quantitative ELISA. We assessed antibody decline using log-linear models. In total, 413 mother-infant dyads were enrolled. At birth, 91.5% (95% CI: 88.8, 94.2) of infants had protective antibody levels, which declined to 26.3% (95% CI: 21.0%, 31.9) at 3 months, 3.4% (95% CI: 0.9, 5.9) at 6 months, and 2.1% (95% CI: 0.1, 4.1) at 9 months. Younger mothers transferred lower levels of antibodies to their infants. We concluded that the majority of infants are susceptible to measles as early as three months of age, suggesting the need to anticipate measles vaccination to protect them.

**Comment**

2) INTRODUCTION:
¬ Newest Global/ Regional/ India/ Chandigarh prevalence of measles cases in children younger than 9 months should be stated.
¬ Risk factors associated with Measles antibodies among infants in Chandigarh, India should be clearly stated.

**Response: We have revised the text extensively as suggested. Global and Indian data have been included. However, there is no previous study reporting measles prevalence and its risk factors in Chandigarh union territory.**

**Revised text:**

Despite the impressive decline in global incidence from 145 cases per million population, to just to 18, during 2000 to 2016, it increased by nearly ten-fold during a global resurgence in 2019 [1]. Recent data suggest that the number of worldwide cases increased by 79 per cent during the first two months of 2022, compared to the preceding year [2]. During 2022, India reportedly contributed the highest number of cases globally [3]. By November 2022, the World Health Organization recorded 12,773 cases, making it the largest outbreak of the year [4]. Despite the impressive decline in global incidence from 145 cases per million population, to just to 18, during 2000 to 2016, it increased by nearly ten-fold during a global resurgence in 2019 [1]. Recent data suggest that the number of worldwide cases increased by 79 per cent during the first two months of 2022, compared to the preceding year [2]. During 2022, India reportedly contributed the highest number of cases globally [3]. By November 2022, the World Health Organization recorded 12,773 cases, making it the largest outbreak of the year [4].

**Comment**

3) METHODS:
¬ There was no clear mention of the questionnaire used. What literature was reviewed to develop the questionnaire? The authors do not provide any references. Was it piloted to assess its internal consistencies? Was it validated? Additional explanations about the validity and reliability of the questionnaire should be added - explained step by step. (Content and structure validity). How long did it take to complete each questionnaire?
¬ Tests of significance should be added.

**Response:**

**We used a simple Case Record Form (CRF) for the enrolled pregnant women (mothers). The CRF comprised questions related to maternal demographics (age, parity, caste, religion, residence, availability of vaccination records, documented prior measles infection, undocumented measles infection). Infant data (date of birth, gestation, birth weight, etc.) were recorded from the hospital records available at birth. During the follow-up, the CRF included questions about symptoms and signs of measles infection in the infant and/or family members (cough, coryza, conjunctivitis, presence of a maculopapular rash, physician confirmation of measles). Data of blood sampling date, and antibody levels, were also recorded.**

**The CRF was prepared in English, and translated to the local vernacular (Hindi and Punjabi). It was pilot tested for ease of use, consistency, and ability to capture the required data. Thereafter, it was finalized and deployed. In the field, each CRF required approximately 10 minutes time for completion.**

**As the focus of this study is not a qualitative field survey (but rather a serial quantification of anti-measles antibody levels), these details were omitted for brevity.**

**Revised text:**

We used a Case Record Form (CRF) to collect data of mothers (age, parity, caste, religion, residence, vaccination records, prior measles infection) and infants (birth date, gestation, weight). During the follow-up, the CRF included questions about symptoms and signs conforming to the clinical definition of measles infection in the infant and/or family members; blood sampling date, and antibody levels. The CRF was prepared in English, and translated to Hindi and Punjabi. It was pilot-tested for ease of use, consistency, and ability to capture the required data. Thereafter, it was finalized and deployed. In the field, each CRF required approximately 10 minutes time for completion.

**Comment**

4) RESULTS:
¬ Table 2 is not clear; it needs more explanation.

**Response: Table 2 presents two sets of analyses. First, a total of ten infant and maternal variables were examined to assess their impact on infants’ antibody levels. For each variable, one category was chosen as the reference (e.g. for infants’ sex, ‘male’ was chosen as the reference). This enabled calculation of exp(β) which is the factor by which the relative risk (of each category vs. the reference category) is multiplied, when the variable increases by one unit.**

**To account for participant attrition over time, the analysis was repeated weighting for the loss to follow-up. This enabled us to assess the impact of participant attrition, on the results. Only two variables retained statistical significance across both analysis ,viz. infants’ age and maternal age.**

**Revised text:**

Table 2 shows results from the multivariable log-linear model with antibody titer as a continuous, log-transformed outcome. A total of ten infant and maternal variables were examined to assess their impact on infants’ antibody levels. For each variable, one category was chosen as the reference, enabling calculation of exp(β) (factor by which the relative risk of each category vs. the reference category, is multiplied, when the variable increases by one unit). To account for participant attrition over time, the analysis was repeated weighting for the loss to follow-up. Only two variables retained statistical significance across both analysis, viz. infants’ age and maternal age.

**Comment**

4) DISCUSSION:
   ¬ Discuss by using the scientific reasoning the anti-measles antibody titers among children younger than 9 months in other developing and developed countries with similar context. The manuscript could be greatly strengthened if the authors could compare the findings of the study with other findings and state the reasons for the strengths and weaknesses in each section.

   [Note: HTML markup is below. Please do not edit.]

**Response: Thank you for the advice. We have added the required text in the Introduction section, to justify the need for this study.**

**Revised text:**

**New text added in the Introduction section:**

Data from different parts of India suggest that 4-12% cases of measles occurred in infants younger than 9 months [8-10]. Analysis of outbreaks in urban slums in Delhi reported significant numbers of cases among infants younger than 9 months, with the youngest case being just one month old [11]. A study in Kerala (southern India), showed that among 43 children with measles, one-third acquired it prior to the age of vaccination [12]. During outbreaks, cases were identified among infants as young as 3-6 months in northern as well as southern India [13-15]. Likewise, an analysis of measles cases among infants younger than 1 year old, showed that 62.5% were younger than 9 months [16]. During an orphanage outbreak in Pune, five of six cases were younger than nine months; four were younger than 4 months old [17].

**New text added in the Discussion section:**

This is not the first study highlighting the problem of infants remaining unprotected from measles prior to the age of vaccination. Besides our own studies highlighted above [21,22], a study in 250 infants showed that only 25 (10%) had IgG antibodies just prior to vaccination [26]. Similarly, another cross-sectional study of 120 infants showed that 85% lacked antibodies prior to vaccination at the age of 9-10 months [27].

Similar experiences have been reported in other countries also. Data from Israel (where measles vaccine is first administered at 1 year of age) showed that less than half of the infants were protected at 6 months of age, and just 4% were protected between 6-11 months of age [28] A study in Turkey examined infants’ antibody levels at one and six months of age. While, 96% of one-month old infants were seropositive, only 25% remained so at 6 months of age, confirming earlier disappearance of maternal antibodies [29].

Unlike the previous cross-sectional studies, our findings are based on a longitudinal cohort of infants. Together, these observations have several implications for public health and policy-making.

5) Reviewers' comments:

   Reviewer's Responses to Questions

**Comments to the Author**

**Comment**

1. Is the manuscript technically sound, and do the data support the conclusions?

The manuscript must describe a technically sound piece of scientific research with data that supports the conclusions. Experiments must have been conducted rigorously, with appropriate controls, replication, and sample sizes. The conclusions must be drawn appropriately based on the data presented.

Reviewer #1: Yes

Reviewer #2: Yes

Reviewer #3: Yes

**Response: Thank you.**

**Comment**

2. Has the statistical analysis been performed appropriately and rigorously?

Reviewer #1: Yes

Reviewer #2: Yes

Reviewer #3: Yes

**Response: Thank you.**

**Comment**

3. Have the authors made all data underlying the findings in their manuscript fully available?

The [PLOS Data policy](http://www.plosone.org/static/policies.action#sharing) requires authors to make all data underlying the findings described in their manuscript fully available without restriction, with rare exception (please refer to the Data Availability Statement in the manuscript PDF file). The data should be provided as part of the manuscript or its supporting information, or deposited to a public repository. For example, in addition to summary statistics, the data points behind means, medians and variance measures should be available. If there are restrictions on publicly sharing data—e.g. participant privacy or use of data from a third party—those must be specified.

Reviewer #1: Yes

Reviewer #2: No

Reviewer #3: Yes

**Response: Thank you for the advice. The dataset has been uploaded.**

**Comment**

4. Is the manuscript presented in an intelligible fashion and written in standard English?

PLOS ONE does not copyedit accepted manuscripts, so the language in submitted articles must be clear, correct, and unambiguous. Any typographical or grammatical errors should be corrected at revision, so please note any specific errors here.

Reviewer #1: Yes

Reviewer #2: Yes

Reviewer #3: Yes

**Response: Thank you.**

**Comment**

5. Review Comments to the Author

Please use the space provided to explain your answers to the questions above. You may also include additional comments for the author, including concerns about dual publication, research ethics, or publication ethics. (Please upload your review as an attachment if it exceeds 20,000 characters)

Reviewer #1: Dear PLOS ONE team of Editorials, thank you for the chance given to me to review “Measles antibodies and susceptibility to disease among infants in Chandigarh, India: A prospective birth cohort study” with manuscript Number PONE-D-22-30962. The study will escalate our knowledge and will update on the antibody of Measles and the susceptibility and its consequent protective rate as well. The following are my comments:

1. We know that the measles vaccine is protective for at least 9 months transferring from mother to child in LMIC and 12 months in high income countries taking in to account e.g., the absence of measles epidemics. Hence, is the study area/ Chandigarh, India/ not measles epidemic affected or epidemic prone area? Why at third, sixth month? You had measured the maternal IgG antibody once. Who measured? How many? What is the qualification to collect the sample? Have you tested the inter-personal reliability? Have you assured quality of both the maternal and newborn biological sample? Do you have SOP? Is that national? Etc

**Response: Thank you.**

1. **Infants in LMICs were believed to be protected from measles till 6-9 months of age, by trans-placentally transferred maternal antibodies. This was based on limited cross-sectional, studies done in infants at various ages, in the pre-vaccination era. As measles was rampant in LMICs in the pre-vaccination era, most mothers had antibodies acquired through natural infection. In contrast, mothers in the current era (who were vaccinated when they were infants) have antibodies acquired through vaccination. Antibodies generated through natural infection are generally of higher titer, and last longer than vaccine-induced antibodies. In the current era, the pattern of antibody decline in infants has not been studied in a prospective cohort, although it is reasonable to expect that the levels would be lower and disappear faster (than among infants whose mothers developed antibodies through natural infection). The findings of this study support this viewpoint.**

**The early disappearance of maternal antibodies makes infants susceptible to measles, a fact which is borne out by witnessing clinical measles among infants younger than 9 months. However, it may be insufficient to create an epidemic.**

2. **India, like most developing countries has a fairly robust universal infant immunization program. Chandigarh union territory has one of the highest vaccination rates in the country. Previously outbreaks have been reported in Chandigarh, although not an epidemic.**
3. **Intervals of 3 months were chosen for infant sampling, so that at least four samples could be obtained prior to the age of vaccination. Less frequent sampling would make it difficult to interpret results. More frequent sampling could provide rich data, but would be burdensome to infants and their family.**
4. **Maternal and infant blood samples were collected by trained nurses who were recruited for the project work. All laboratory testing was performed at PGIMER by a trained laboratory technician recruited for the Project, and in accordance with the National Accreditation Board for Testing and Calibration Laboratories (NABL) Standards, ISO 17025 for public health laboratories, as well as the Manual for the laboratory diagnosis of measles and rubella, 2nd edition (WHO/IVB/07.01), WHO, Geneva, Switzerland. Quality system essential elements identified in CLSI GP26A3, that specifically apply to this project met these standards.**

**Revised text: The following sentences have been added (at different places) in the revised manuscript.**

All blood sampling was performed by trained nurses who were hired for the study.

Three-month intervals were chosen to optimize the number of samples per infant, without over-burdening the infants or their families

Laboratory testing was performed by a trained laboratory technician in accordance with the National Accreditation Board for Testing and Calibration Laboratories (NABL) Standards, ISO 17025 for public health laboratories, as well as the World Health Organization Manual for the laboratory diagnosis of measles and rubella, 2nd edition (WHO/IVB/07.01).

**Comment**

2. Why you choose to conduct there? What is unique about the study area? The pre-pregnancy and pregnancy characteristics of the respondents and additional characters should be described.

**Response: Chandigarh union territory has a well-defined geographic area, with a well-documented population neatly categorised into urban, rural, and urban-resettlement colony (formerly called urban slum) areas. Additionally, Chandigarh has a total of 510 well-functioning anganwadi units, that cater to the basic health needs of a pre-specified population (in particular pregnant mothers and pre-school children). Third, Chandigarh has not been involved in large-scale intervention studies that could alter the basic epidemiology of infectious diseases. Last, Chandigarh has a fairly high coverage with routine childhood vaccines, and a robust system of recording infant vaccination. For these reasons, it is an ideal area to study the epidemiology of measles.**

**Revised text: None**

**Comment**

3. It is longitudinal study with repeated measurements why you prefer longitudinal analysis over others?

**Response: It is well known that prospective, longitudinal, cohort studies are superior to cross-sectional studies at multiple time points. Performing cross-sectional studies of infants at different ages, would not permit a correlation between their maternal antibody levels at delivery, and the dynamics of waning of the maternally transferred antibodies.**

**Revised text: None**

**Comment**

4. The outcome variable is the measles susceptibility and protection status of the maternal transferred anti-body against measles? Re-write the title again.

**Response: Thank you for the suggestion. The title has been revised as follows.**

**Revised text:**

**Dynamics of maternally transmitted anti-measles antibodies and susceptibility to disease among infants in Chandigarh, India: A prospective birth cohort study.**

**Comment**

5. The selection of the women and the newborns should be revisited. E.g., If mothers give town newborn, how did you manage?

**Response: We respectfully state that this question is unclear. As stated in the Methods section:**

**Pregnant women residing within the 30 anganwadi clusters were approached during the third trimester of pregnancy, by research personnel to explain (in the local vernacular) about the study, its purpose and procedures. The pregnant women who were willing to participate in the study were enrolled at the time of delivery, and their infants were enrolled immediately after delivery. Mothers with a health condition that hindered normal daily activities; or who had acute febrile illness, hemophilia, or any other blood disorder that could lead to excessive bleeding, were excluded. Infants were excluded if they required resuscitation at birth or had any life-threatening congenital anomaly. The mother-infant dyads comprised the study population. Infants were followed longitudinally at 3, 6, 9, and 12 months of age. Chandigarh has a fixed number of institutions where pregnant women deliver babies. The PI’s institution caters to the bulk of the deliveries.**

**Revised text: Please advise.**

**Comment**

6. What is the prevalence and rate Measles in the study area?

**Response: There is no robust, population data on the prevalence and rate of measles in the study area (Chandigarh) available in the public domain. However, the area has witnessed periodic outbreaks, three of which have been published (please see the citations below). Based on this, the Department of Virology at the study institution (PGIMER Chandigarh) was designated a nodal laboratory for measles outbreak response.**

**Thakur JS, Ratho RK, Bhatia SP, Grover R, Issaivanan M, Ahmed B, Parmar V, Swami HM. Measles outbreak in a Periurban area of Chandigarh: need for improving vaccine coverage and strengthening surveillance. Indian J Pediatr. 2002 Jan;69(1):33-7.**

**Sharma MK, Bhatia V, Swami HM. Outbreak of measles amongst vaccinated children in a slum of Chandigarh. Indian J Med Sci. 2004 Feb;58(2):47-53**

**Ratho RK, Mishra B, Singh T, Rao P, Kumar R. Measles outbreak in a migrant population. Indian J Pediatr. 2005 Oct;72(10):893-4.**

**Revised text: Please advise if the above should be inserted into the manuscript.**

**Comment**

7. What is the ICC level and why you prefer auto-regressive covariance over others?

**Response: In general, autoregressive covariance is appropriate when the correlation between responses decays over time, as in this study. An unstructured covariance is generally used when the variance is heterogenous.**

**Revised text: Please advise if this needs to be inserted into the manuscript.**

**Comment**

8. The recommendation should be drawn from your findings. E.g., the recommendation should be RCT of the Measles anti body study transfer and the susceptibility and protection of Newborns from measles infection.

**Response: Thank you for the advice. The Conclusion has been modified as shown below.**

**Revised text:**

**Discussion section:**

However, determining the optimal age of infant vaccination would require well-designed randomized controlled trials, with adequate follow-up and meticulous monitoring to ensure that infants are adequately protected against measles.

**Conclusion section:**

This longitudinal birth-cohort study identified that the majority of infants were susceptible to measles, well before the age of vaccination. This suggests the need to protect them either through anticipated vaccination schedules, or enhancing the antibody levels in pregnant women. Meanwhile, we recommend strengthening of surveillance to determine if these susceptible infants develop clinical or subclinical measles.

**Comment**

9. Tables are not self-explanatory, sentences are incomplete, revisit the language and the sentence again.

**Response: Thank you for the advice. The Tables and associated text have been revised.**

**Revised text:**

**Please see the revised tables and text.**

**Comment**

10. Check and consult for the statistics.

**Response: Thank you for the advice. The statistical analysis has been rechecked and confirmed.**

**Comment**

Reviewer #2: The topic of interest is relevant and appropriate. However,
1. Why was the study published after a gap of 4 yrs?

**Response: We acknowledge a delay in manuscript preparation and submission. The investigators worked on other manuscripts related to the study in the interim.**

**Comment**

2. What is the role of maternal educational status on the antibody titres?

**Response: In general, infant vaccination rates in developing countries (including India) are higher when mothers are better educated. Therefore, maternal education is always included as a variable in studies of infant immunization and immunity.**

**Maternal education levels could indeed impact their antibody levels. Better education is an indirect marker of women empowerment, and societal structure. Better educated mothers tend to hail from families with better socio-economic backgrounds, and greater emphasis on woman independence. In such a situation, better educated mothers are more likely to have been vaccinated against measles when they were infants. This could account for relatively lower titres of antibodies in them, and hence lower levels in their infants.**

**On the other hand, limited data suggest that the reverse is also possible. A case-control study in China identified that low education level of the primary caregiver was associated with a higher risk of measles among infants younger than 8 months [*Tang ZZ, Xie YH, Jiraphongsa C, Liu XH, Li ZY, Chongsuvivatwong V. Risk factors for measles in children younger than age 8 months: A case-control study during an outbreak in Guangxi, China, 2013. Am J Infect Control. 2016 Apr 1;44(4):e51-8*.].**

**However, as the study data did not conclusively prove that maternal education was inversely related to infant antibody levels, we did not highlight this point.**

**Comment**

3. What is the incidence of measles cases in infants under 9 months?

**Response: There is limited robust data on the burden of measles cases among infants younger than 9 months. However, data from different parts of India suggest that 4-12% cases of measles occurred in infants younger than 9 months [8-10]. Analysis of outbreaks in urban slums in Delhi reported significant numbers of cases among infants younger than 9 months, with the youngest case being just one month old [11]. A study in Kerala (southern India), showed that among 43 children with measles, one-third acquired it prior to the age of vaccination [12]. During outbreaks, cases were identified among infants as young as 3-6 months in northern as well as southern India [13-15]. Likewise, an analysis of measles cases among infants younger than 1 year old, showed that 62.5% were younger than 9 months [16]. During an orphanage outbreak in Pune, five of six cases were younger than nine months; four were younger than 4 months old [17].**

**This information has been incorporated into the revised text as follows:**

**Revised text:**

Data from different parts of India suggest that 4-12% cases of measles occurred in infants younger than 9 months [8-10]. Analysis of outbreaks in urban slums in Delhi reported significant numbers of cases among infants younger than 9 months, with the youngest case being just one month old [11]. A study in Kerala (southern India), showed that among 43 children with measles, one-third acquired it prior to the age of vaccination [12]. During outbreaks, cases were identified among infants as young as 3-6 months in northern as well as southern India [13-15]. Likewise, an analysis of measles cases among infants younger than 1 year old, showed that 62.5% were younger than 9 months [16]. During an orphanage outbreak in Pune, five of six cases were younger than nine months; four were younger than 4 months old [17].

**Comment**

Reviewer #3: There is the need for the authors to show how they arrived at the sample size in a more explicit manner. The relevance of the essence of collecting data on religion and cast has not been optimally utilised to arrive at a conclusion on whether these have any influence on the results obtained. There is the need to explain more about this in the discussion section. These could be related to the attitude and practice of the people towards vaccination or be responsible for susceptibility to infection.

**Response: Thank you for the advice.**

**Sample size calculation**

**This has been explained in more detail. Please see the revised text.**

**Relevance of collecting data on caste and religion**

**In India (and in many developing countries), the socio-demographic factors affecting infant vaccination rates, susceptibility to infectious diseases and access to healthcare (in general) include: socio-economic status, maternal education, caste, religion, and markers of woman empowerment (maternal age, parity). We have previously shown that the odds of seroconversion following measles vaccination was 0.78 (95% CI: 0.62, 0.98) among infants from Scheduled Castes/ Scheduled Tribes compared to the ‘Others’ caste group [*Wagner AL, Mathew JL, Carlson BF, Kachoria AG, Bharti B, Suri V, Boulton ML. Measles Vaccination Immunogenicity and Association with Caste in Chandigarh, India. Am J Trop Med Hyg. 2022 Oct 3;107(5):1129-1131.].***

**However, in this analysis, there was no statistically significant impact of family religion or caste on the infant antibody levels, hence we have not emphasized it.**

**Revised text:**

We collected data on several additional variables that impact infant vaccination and susceptibility to disease, but none showed statistically significant impacts on anti-measles antibody levels.

Was there any difference in the data obtained in rural, urban and resettlement colonies of Chandigarh? This was not reflected in any way in the discussion. What was the relevance of the anthropomorphic measurements?

**Response:**

**We did not examine the data by the type of residence, hence are unable to assess its impact of any. We will conduct this analysis and report it in a separate publication.**

**In developing countries, malnourished infants generally fare worse in terms of vaccine-induced immunity and infectious disease burden. Therefore,, these data are routinely collected in research studies. We plan to undertake additional analysis to study the impact of nutritional status (if any).**

6. PLOS authors have the option to publish the peer review history of their article ([what does this mean?](https://journals.plos.org/plosone/s/editorial-and-peer-review-process#loc-peer-review-history)). If published, this will include your full peer review and any attached files.


If you choose “no”, your identity will remain anonymous but your review may still be made public.


**Do you want your identity to be public for this peer review?** For information about this choice, including consent withdrawal, please see our [Privacy Policy](https://www.plos.org/privacy-policy).

Reviewer #1: No

Reviewer #2: No

Reviewer #3: No
